# Q Fever Endocarditis in Northeast Iran

**DOI:** 10.1155/2021/5519164

**Published:** 2021-05-28

**Authors:** Ali Akbar Heydari, Ehsan Mostafavi, Masoumeh Heidari, Mina Latifian, Saber Esmaeili

**Affiliations:** ^1^Research Center for Infection Control & Hand Hygiene, Mashhad University of Medical Sciences, Mashhad, Iran; ^2^Department of Epidemiology and Biostatics, Research Centre for Emerging and Reemerging Infectious Diseases, Pasteur Institute of Iran, Tehran, Iran; ^3^National Reference Laboratory for Plague, Tularemia and Q Fever, Research Centre for Emerging and Reemerging Infectious Diseases, Pasteur Institute of Iran, Akanlu,Kabudar-Ahang, Hamadan, Iran

## Abstract

This report presents a case of chronic Q fever endocarditis. A 60-year-old male farmer and rancher was admitted to the hospital with symptoms of weight loss, fever, severe sweating, weakness, and anorexia. PCR was negative for *C. burnetii* in the blood sample, but phase I and II IgG antibodies against *C. burnetii* were positive (1 : 16384 and 1 : 2048, respectively) by the indirect immunofluorescent assay (IFA). According to the adjusted Duke criteria, Q fever endocarditis was confirmed, and the patient was successfully treated with doxycycline and hydroxychloroquine.

## 1. Introduction

*Coxiella burnetii* is an obligate intracellular bacterium that infects mononuclear phagocytes and causes a zoonotic disease called Q fever. Domestic animals (especially, cow, goat, and sheep) are the main reservoirs of *C. burnetii*. *C. burnetii* infection in humans is mainly acquired by inhalation of *C. burnetii*-contaminated aerosols shedding from infected animals [1]. Q fever is manifested in both acute and chronic forms in humans. The clinical presentation of acute Q fever is nonspecific. Chronic Q fever can occur early or late after acute Q fever and be a primary manifestation of Q fever. The main clinical manifestation of chronic Q fever is endocarditis, which, if not treated, can lead to heart failure and death [2].

Q fever has a worldwide distribution. The disease is endemic in Iran [3, 4]. In recent years, acute Q fever cases were reported in different studies in Iran: the first Q fever endocarditis was reported in 2014 [5]. In 2019, sixteen patients with Q fever endocarditis were diagnosed among culture-negative endocarditis cases [6]. However, it seems that the disease is not yet taken into account by the healthcare system and clinicians in Iran. In this report, a case of culture-negative Q fever endocarditis is presented.

## 2. Case Presentation

The patient was a 60-year-old male farmer and rancher who lived in the rural region of Chenaran County in Khorasan Razavi province (northeast Iran). The patient had a history of direct contact with domestic animals such as sheep and goats. On 21 March 2018, his symptoms were started with mild fever (which had no specific pattern) and severe night sweats. Also, chills, weakness, lethargy, and fatigue were gradually added to the clinical symptoms. During the next five months, he was referred to different physicians for treatment, but the patient's condition did not improve.

The patient was hospitalized on 8 July 2018, with a complaint of fever, severe night sweats, weakness, lethargy, severe fatigue, severe anorexia, and weight loss (4.5 kg during the last five months). The patient had no history of immune deficiency. At the initial examination, SpO_2_, temperature, and blood pressure were 94%, 38°C, and 130/80 mmHg, respectively. In the cardiac examination, there was a systolic murmur (grade: 3/6), the heart rhythm was irregular, and the pulses were full, symmetrical, and irregular. No fingertip cyanosis and clubbing were observed. No abnormalities were reported on abdominal examination.

Liver enzyme tests, electrolytes, blood urea nitrogen, and creatinine were normal. Other tests were performed to monitor the patient symptoms, including urine test, coagulation test, and rheumatoid test, all of which were reported to be normal. WBC count (8000 cells/*μ*l), platelet, hemoglobin level, and erythrocyte sedimentation rate (24 mm/h) were normal, but the C-reactive protein level was increased (32.3 mg/dl).

Increased vascular marking was evident on chest radiographs. Otherwise, it was normal. On echocardiography performed for the patient, the right and left ventricles were enlarged, and a highly mobile and hypovascular mass with 1*∗*7*∗*9 mm size was reported to be attached to the atrial side of the mitral valve. According to clinical and paraclinical and echocardiographic findings, the probable diagnosis of endocarditis was suggested for the patient. Blood cultures were negative several consecutive times, and the results of serological and culture tests for brucellosis were negative. Finally, due to the clinical course of the disease and the negative results of blood cultures, attention was drawn to culture-negative endocarditis, especially Q fever. In order to confirm the diagnosis of Q fever, blood and serum samples of the patient were sent to the National Reference Laboratory of Q Fever in Pasteur Institute of Iran, and serological and molecular tests were requested.

PCR was negative for the detection of *C. burnetii* in the blood sample. Phase I and II IgG antibodies against *C. burnetii* were evaluated by the indirect immunofluorescent assay (IFA) (Focus Diagnostics, Cypress, CA, USA), and antibody titers of IgG phase I and II against *C. burnetii* were 1 : 16384 and 1 : 2048, respectively. According to these results, Q fever endocarditis was considered to be the final diagnosis.

From the time of hospitalization until the time of suspected culture-negative endocarditis, the patient was treated with ceftriaxone 1 gr/day. Afterwards, due to the probable diagnosis of culture-negative endocarditis, the previous drug was changed to ciprofloxacin (400 mg twice daily), ampicillin-sulbactam (3 gr every 6 hours), and gentamicin (240 mg/daily). After the confirmation of Q fever endocarditis, doxycycline (100 mg/ twice daily) and hydroxychloroquine (200 mg every 8 hours) were prescribed for 18 months. According to the echocardiographic findings and in consultation with the cardiac surgery team, the patient became a candidate for angiography and mitral valve repair, but the patient refused surgery. The patient was also visited every three months for 8 months and was found to be well and satisfied over the follow-up period. After that, the patient did not continue the regular contact with us.

## 3. Discussion

In this report, the first case of chronic Q fever endocarditis was presented from northeast Iran. The conversion rate of acute to chronic Q fever ranges from 0 to 5% [2]. Complications of Q fever are found in about 60% of those with chronic disease. The mortality rate in the chronic form is about 38%, and endocarditis, infected aneurysm, and vascular prosthesis are the most common forms of infection [7]. Based on recent evidence, serological follow-up of all known acute Q fever patients at least once during the first year following the acute infection and more frequently in patients with known risk factors for chronic diseases, such as heart valve or vascular prosthesis, is very critical [8]. Therefore, all culture-negative endocarditis cases must be evaluated according to the Duke diagnostic criteria for chronic Q fever, and any failure to diagnose leads to death [2].

In northeast Iran, there have been reports of livestock infection and contamination of dairy products by *C. burnetii* in recent years [9, 10]. Also, acute Q fever prevalence was reported 7.4% in acute undifferentiated febrile illnesses by PCR in Khorasan Razavi province [11]. This patient was the first case of Q fever endocarditis in northeast Iran. Despite the evidence that shows the prevalence of *C. burnetii* in humans and livestock in this region, the health system and clinicians less notice Q fever endocarditis.

The patient who we reported was a farmer. After the onset of symptoms and referral to the hospital, the patient was reported as having culture-negative endocarditis and examined for chronic Q fever. Based on echocardiographic findings and mitral valve tissue damage, molecular and serological tests were performed for him. Antibody titers of IgG phase I and II against *C. burnetii* by IFA were 1 : 16384 and 1 : 2048, respectively. The IFA method is one of the best and most approved methods for detecting phase I and II antibodies against *C. burnetii*. In fact, IFA is the gold standard and the first-line diagnostic technique for the diagnosis of Q fever patients [2]. Also, due to the use of a wide range of antibiotics by the patient, the molecular tests were negative in our case.

Various studies in Iran have shown that Q fever has a significant seroprevalence in both humans and animals [3], and also, the prevalence of *C. burnetii* has been very high in milk and abortion samples of domestic animals [4, 12]. Therefore, Q fever should be considered as one of the most important and endemic diseases in Iran. On the contrary, the exact prevalence of Q fever endocarditis has not been fully determined in Iran, and extensive and multicenter studies are needed to make the right judgment about it. It seems that Q fever is one of the important causes of the culture-negative endocarditis in Iran [6], and much attention should be paid to this disease in Iran by the healthcare system and clinicians.
